# Investigations of Bread Production with Postponed Staling Applying Instrumental Measurements of Bread Crumb Color

**DOI:** 10.3390/s91108613

**Published:** 2009-10-28

**Authors:** Jovanka V. Popov-Raljić, Jasna S. Mastilović, Jovanka G. Laličić-Petronijević, Vladimir S. Popov

**Affiliations:** 1 Faculty of Agriculture, University of Belgrade, Nemanjina 6, Belgrade-Zemun, Serbia; E-Mails: jpopov@agrif.bg.ac.rs (J.V.P.-R.); jovankal@agrif.bg.ac.rs (J.G.L.-P.); 2 Institute for Food Technology, University of Novi Sad, Bulevar Cara Lazara 1, Novi Sad, Serbia; 3 Faculty of Agronomy, University of Novi Sad, Trg Dositeja Obradovića 8, Novi Sad, Serbia; E-Mail: vladapopov78@gmail.com (V.S.P.)

**Keywords:** flour, bread, different compositions, bread staling, color measurement, storage

## Abstract

Crumb color quality characteristics of bread of different compositions (whole grain, rye, barley and diet bread) at 24 hours intervals during three days after bread preparation were investigated by means of a MOM-color 100 tristimulus photo colorimeter, in CIE, CIELab, ANLAB and Hunter systems. The highest value of average reflectance *y* (%) was found for barley bread (immediately after preparation), so that can be said that this sample was “conditionally” the lightest. The lowest values of *y* (%) were found for diet bread, so that it can be considered as the “conditionally” the darkest product. Colors of all investigated bread samples were lighter after three days of keeping compared to day 0. Changes of average reflectance of bread samples packed in polyethylene packaging with keeping time can be described by linear equation (correlation coefficient 0.99). The dominant wavelength of barley and diet bread confirm the presence of yellow pigment. Color qualities of the mentioned kinds of bread depend on processes during bread staling and raw material composition of bread (flour). Color quality measurements can be used as easy auxiliary method for screening in the development of slower staling bread.

## 1. Introduction

Color of a product is the visual effect of subjective sensation of an observer [1,2]. Every colored substance selectively absorbs light of visual part of spectrum. When white sunlight shines upon a colored molecule, a certain part of the light spectrum is absorbed while the other is reflected and creates the impression of color in the observer's eye. The colored light beam which reaches the eye is in the range of wavelengths of dominant colors, and the intensities of particular wavelengths are variable. Pure colors, defined with only one wavelength, do not exist. Each color is characterized by the sum of wavelengths of different intensities and can be presented as a dominant wavelength. Brightness is an optical quality of color and presents a measure of intensity of the color sensation. Brightness means the average reflectance and it is classified by the range in which white color has the highest and black color the lowest value; e.g., higher values of average reflectance represent higher brightness and lower values, darker color, mean lower brightness. The increase of brightness defines darkening of the product [3,4]. The Commission International de l'Eclairage (CIE) has suggested the CIE color system for the instrumental determination of food color [5]. This system is based on a “standard eye” with filters for three primary colors: red, green and blue [6,7]. Color characteristics are determined through dominant wavelength λ (nm) and average reflectance *y* (%).

Quality of bread, as the final product, depends on the quality of raw materials (flours) and additives, as well as on the characteristics of the production process applied. Lamsal and Faubion [8] investigated the influence of enzyme preparations on color of wheat flour, dough, mixing and baking tests and stated that the enzyme preparation was efficient with respect to wheat flour and dough whitening, especially if it was added prior to the wheat milling. The literature data include also the results on bread crumb color changes (Hunter Lab system) caused by a proportion of wheat flour (10–30%) with the banana flour [9], or with the defatted corn germ flour [10], where it was stated that all investigated physical and sensory bread quality parameters were highly correlated with the measured bread crumb color parameters.

Different cereal components contribute to specific taste of the final product affecting the human health at the same time [11-14]. With the corresponding composition of initial raw materials and at the same time a good technological process, it is possible to obtain the final product—bread or bread-like products designed for special categories of consumers (such as, for example, diet bread designed for diabetics [15]. Crumb color depends on flour color, which, on the other hand, depends on grain endosperm color and on quantity and color of peripheral flour particles (bran) [16,17]. Flour granulation and content of pigment substances in the mealy endosperm also affect flour color [18]. Investigating the flour color quality, Oliver *et al.* [19] found that the value of psychometric light was in correlation with contents of mineral substances, while the value of psychometric hue was in correlation with yellow pigment content. Aquistucci and Pasqui [20] concluded that the extracted part of wheat (grits), which is independent of ash content of flour, affects the measurement of psychometric light level.

Sensory evaluation of bread is a very “hard” and sensitive task, often with contradictory results, even with the highly experienced and trained sensory panel and especially for the crumb color evaluation in the case of minor formulation and processing changes [21]. Because of that, it is very desirable to apply instrumental, reproducible and sensitive crumb color measurement.

## 2. Material and Methods

### 2.1. Bread Baking Procedure

Crumb color qualities of different kinds of bread were investigated. The compositions of the samples were (% on flour):

1. *Whole meal bread*: whole grain flour, 100; baker's yeast, 3.0; salt, 1.7; margarine, 3.0; caraway seeds, 0.15;
2. *Rye bread*: wheat flour T-950, 60.0; rye flour T-950, 40.0; baker's yeast, 4.0; salt, 2.0; additive, 0.4; caraway seeds, 0.3;
3. *Barley bread*: wheat flour T-950, 80.0; barley flakes, 20.0; baker's yeast, 3.5; salt, 2.0; margarine, 1.0; additive, 0.2; and
4. *Diet bread*: wheat flour T-950, 85.0; bran, 15.0; baker's yeast, 3.0; salt, 2.0; additive, 0.4.

Bread samples were mixed and baked in the experimental bakery of the Institute for Food Technology of the University of Novi Sad (in total about 400 bread samples were used). In brief, the production procedure was as follows: mixing with the two-speed rapid Diosna mixer, 5 minutes at 85 rpm and 3 minutes at 120 rpm. Fermentation 60 + 30 minutes, with manual intermediate dough kneading. Machine loaf forming; proofing till the optimal dough condition (t = 30 °C throughout the dough mixing process). Baking in the MIWE oven, 230 °C, 25 minutes.

After baking, bread samples were cooled down in chambers with conditioned air atmosphere, and then packed in the corresponding packaging material (PE, 20 μm) and kept, i.e., storaged at ambient temperature (20 ± 2 °C).

### 2.2. Instrumental crumb color measurements and calculations

Characteristics of crumb color qualities were determined by tristimulus photo colorimeter MOM-color 100 in the CIE, CIELab, ANLAB—Adams Nickerson's and Hunter systems, based on 20 independable measurements for each point for each sample and each day of storaging (in total 320 different bread samples).

Average reflectance or brightness, i.e., darkening of color *y* (%) is normally measured, and changes of dominant wavelengths (nm) are followed.

In the CIE-system, the dominant wavelengths of investigated samples are determined on the basis of values *x*_1_, *x*_2_, *y* and *z*, obtained on tristimulus photo colorimeter MOM-color 100. The tristimulus coefficients *X* and *Y* are further calculated by the use of formula:

(1) $$X=\frac{x}{x+y+z}\;\text{and}\;Y=\frac{y}{x+y+z}$$

with: *x* = *x*_1_ + *x*_2_.

**Figure 1.**
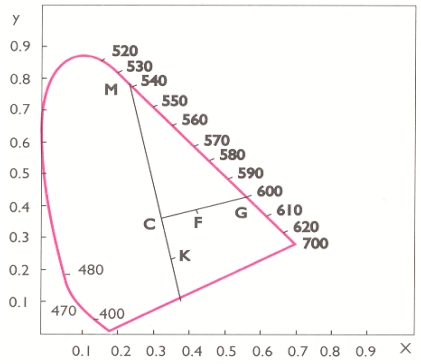
Determination of dominant wavelength and purity of color by CIE system.

These *X* and *Y* values in the chromaticity diagram [5,22,23] determine point *F*, which is connected with point *C*, which is obtained by calibration of the photo colorimeter with standard. The *CF* line is prolonged and its intersection with contours of diagram (Figure 1) determines the dominant wavelength.

Apparatus itself is calibrated with the standard white “observer”, which is characterized with following tristimulus values: *x*_1_ = 63.21; *x*_2_ = 15.81; *y* = 81.28 and *z* = 95.01, and can be perceived as the “standard eye” with filters for standard colors (red, green and blue) [24].

Calculated trichromatic coefficients are used for calculation of the color purity in percents, i.e., for reading off of dominant wavelength, on the base of the chromacity diagram. Dominant wavelength (λ) is determined on the basis of the calculated trichromatic coefficients, which are introduced into the chromacity diagram (point F), which is to be jointed with the point C, and extender to the intersection with the spectral curve. Point of intersection [point G represents the dominant wavelength (Figure 1)].

Color purity is expressed in percents and it is to be calculated on the basis of the following relation of points:

(2) $$\text{color purity}\;(\%):P=\frac{\bar{CF}}{\bar{FG}}\cdot 100$$

In the CIE system, average reflectance or brilliance is determined on the basis of the *y* (%)—value which is read out directly at the MOM—color.

In the CIELab system, color quality characteristics are expressed on the basis of the following equations:

(3) $$\text{psychometric light}:L^{*}=116\cdot {\left(\frac{Y}{Y_{0}}\right)}^{\frac{1}{3}}-16$$

(4) $$\text{psychometric tone}:a^{*}=500\cdot \left[{\left(\frac{X}{X_{0}}\right)}^{\frac{1}{3}}-{\left(\frac{Y}{Y_{0}}\right)}^{\frac{1}{3}}\right]$$

(5) $$\text{psychometric chroma}:b^{*}=200\cdot \left[{\left(\frac{Y}{Y_{0}}\right)}^{\frac{1}{3}}-{\left(\frac{Z}{Z_{0}}\right)}^{\frac{1}{3}}\right]$$

a*—psychometric tone: [participation of red (+) and green (−) colors of components];

b*—psychometric chrome [participation of yellow (+) and blue (−) colors of components].

Color difference with respect to the standard white, according to Robertson [4], is defined as:

(6) $$\Delta H_{ab}^{*}=C^{*}\cdot \Delta h^{*}\frac{\pi }{180}$$

According to Hunter [25], the mentioned values are calculated on the base of the following equations:

(7) $$\text{psychometric light}:\;L_{Hu}=100\cdot \sqrt{\frac{Y}{Y_{n}}}$$

(8) $$\text{psychometric tone}:\;a_{Hu}=K_{a}\cdot \left[\frac{\frac{X}{X_{n}}-\frac{Z}{Z_{n}}}{\sqrt{\frac{Y}{Y_{n}}}}\right]$$

(9) $$\text{psychometric chroma}:\;b_{Hu}=K_{b}\cdot \left[\frac{\frac{Y}{Y_{n}}-\frac{Z}{Z_{n}}}{\sqrt{\frac{Y}{Y_{n}}}}\right]$$

where*X, Y, Z* represent CIE tristimulus values,*X_n_, Y_n_, Z_n_* are tristimulus values taken from tables connected with the light source,*K_a_, K_b_* are coefficients of chromaticity for the light source, and*Y_n_* = 100.00—for each occasion.

For determination of degree of difference of color between sample and the standard white, there exists possibility of calculation of Δ*E_Hu_*-values:

(10) $$\Delta E_{Hu}=\sqrt{{\left(\Delta L_{Hu}\right)}^{2}+{\left(\Delta a_{Hu}\right)}^{2}+{\left(\Delta b_{Hu}\right)}^{2}}$$

Colors of samples were determined immediately after their preparation, and after 1, 2 and 3 days of storage in polyethylene packaging (20 μm) at room temperature (20 ± 2 °C). Changes of average reflectance with keeping time are described using an appropriate mathematical model.

In scopes of the instrumental determinations of characteristics of color measurement, the equation of trend the best adapted to the average reflectance (*y*, %) changes was obtained with the statistical software Origin 6.1 (Origin Lab. Corporation, Northampton, MA, USA).

## 3. Results and Discussion

Color quality characteristics of crumb of different kinds of breads immediately after their production (day 0) obtained by using all four known instrumental color measurement systems (CIE, CIELab, ANLAB—Adams Nickerson's and Hunter's systems) are presented in the Table 1. Similar measurements were performed after 1 and 2 days of bread storage in the polyethylene packaging (data not shown), as well as after 3 days of storage (Table 2). The highest average reflectance was found in barley bread (*y* = 48.99% after the preparation and *y* = 55.18% after 3 days), so that it can be accepted as the lightest product.

Similar conclusions were observed during determination of color quality characteristics for samples of the barley bread by the CIELab system. Immediately after processing value of psychometric light amounts to *L*= 75.44, and after three days it was *L* = 79.14; at the same time values of psychometric tone were *a* = −7.79 and *a* = −33.84, and values of psychometric chroma *b* = 25.11 and *b* = 23.01. The corresponding values in the ANLAB system were *L* = 67.74 and *L* = 72,27; *A* = −7.43 and *A* = 32.47; *B* = 28.83 and *B* = 21.98; Hunter's system gave values of *L*_Hu_ = 69.99 and *L*_Hu_ = 74.28, *a*_Hu_ = −7.08 and *a*_Hu_ = −29.57, and *b*_Hu_ = 19.84 and *b*_Hu_ = −29.57) (Tables 1 and 2).

The lowest values of average reflectance (*y* = 30.85% after the preparation, and *y* = 39.83% after 3 days) were determined in diet bread. Immediately after production, the psychometric light value of diet bread samples was *L* = 62.38, and after 3 days of storage it was *L* = 62.96. At the corresponding times, shares of green pigment components, i.e., of psychometric tone were *a* = −9.59 and *a* = −29.00, while respective values of psychometric chroma (share of yellow pigments) were *b* = 38.36 and 25.08 (Tables 1 and 2).

Crumb color depends primarily on the type of flour. Flour containing coarsely ground bran results in lighter crumbs compared to flour with finely ground bran. Fine bran is uniformly homogenized with flour, and it makes baked bread crumbs darker. The finer the crumb grain structure results in a lighter color, and conversely, arger crumb pores induce darker color. Differences in crumb color are due to the shadows on the walls of holes [26]. Rye bread is not very porous, as there is no gluten in the flour and gases cannot be retained in the dough. In contrast to white bread, rye bread is slower drying, due to different amounts of water bound in the crumbs. Therefore, the average reflectances of barley- and rye breads are relatively the equal, *y* = 55.18% and *y* = 54.35%, respectively. Wholemeal bread was made of 100% whole grain flour, e.g., semolina. Whole meal is very “rough” flour containing rough parts of grain which cause somewhat darker crumbs. Diet bread contains 15% of finely ground bran distributed in white flour. The results are darker color and more voluminous pores. After three days of storage, colors of all samples were lighter, with higher average reflectance compared to samples after preparation. Medium with higher moisture content or where the whole amount of water is not bound, is darker than the drier one. Storage of bread for three days resulted in color changes, although samples were packed in appropriate packaging material. Evidently, during storage of packaged bread samples, (so called “permanent” bread), bread does not behave as a “dead” substance, but it undergoes permanent changes, known as staling, to the higher or lower extent, that reflect in the bread crumb color parameters.

**Figure 2.**
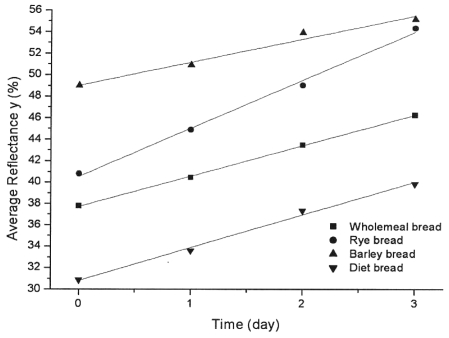
Experimental and calculated average reflectance values.

**Figure 3.**
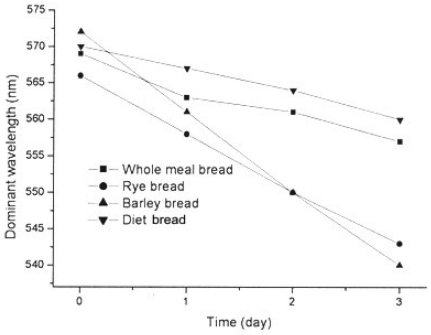
Dominant wavelength changes of bread samples.

Having this in mind, we found that it could be of interest to examine the bread color changes with the duration of storage. It was found that, irrespective of the color parameter chosen or color measurement system (CIE, CIELab, ANLAB—Adams Nickerson's and Hunter's systems) applied, this changes were linear, with parameters (slopes *a* and Y-axis intercepts *b*), that were dependent of the bread kind, i.e., on its raw materials composition, technological process of production, storaging conditions and packaging. As the examples, this was shown for the average reflectances *y* (%) (Figure 2), and for the dominant wavelength λ (nm) in the CIE system (Figure 3).

Experimental values of average reflectance and the fitted curve describing the dependence of average reflectance and keeping time of bread are presented in Figure 2. The figure and the correlation coefficient (0.99) given in Table 3, show that the equation for the straight line describes very well the changes of average reflectance with time, for all bread samples. Although samples were packed in polyethylene packaging for three days at room temperature, staling of samples resulted in crumb color quality, e.g., average reflectance, changes. Due to the bread staling and its crumb changes, crumb colors gradually became lighter, e.g., the average reflectances increased. The obtained results show that this process follows a linear change.

According to the slope of fitted straight line for different bread samples, staling of rye bread was the fastest, followed by whole meal-, and by barley bread. This finding confirmed the mentioned statements that the bread staling parameters depend on, raw material composition of bread (flour), but also of the applied accessory raw materials (additives) as well as on the technological process of bread manufacturing. The intercept value on the ordinate in 0 day (usually designed with *b*) for the investigated samples, confirms the visual conclusion after the preparation of bread, namely, that the diet bread was the darkest, somewhat lighter was the whole grain bread, followed by rye bread, and barley bread at the end.

Changes of dominant wavelength with time of keeping are given in Figure 3. The highest values of dominant wavelength (after the preparation) were found in barley bread samples, pointing to the presence of yellow pigment. Decreases of dominant wavelength were noticed in all samples, however, they were the most expressed in barley and rye bread samples. It is the consequence of raw material composition of bread (flour).

A number of factors affect bread crumb staling such as: enzymatic additives, addition of acids (ascorbic, lactic), time of dough mixing, leveling degree, duration and temperatures of dough of baking and bread cooling [17,26,27]. All these factors reflect themselves on changes of the bread crumb color parameters. With that in mind, it is possible to assume that the changes of the bread color parameters for the postponed bread staling should be as low as possible. In the other words, the slopes of lines showing their changes with the keeping time should be as low as possible.

Instrumental bread crumb color measurements (using, for example tristimulus photoelectric colorimeters) are fast, easy, reproducible and inexpensive. So, it is logical to suppose that they could be used as a starting base for development of new formulations of breads with the slower staling characteristics, i.e., for the preliminary screening of a huge numbers of samples of process variables or variants. After such screenings and choosing up of a reasonable number of variants, these variants could be finally evaluated by application of expensive and time consuming chemical and/or rheological procedures and sensory evaluations aimed to the obtaining of a new bakery products with the retarded staling and the prolonged shelf life [10].

## 4. Conclusions

Crumb color quality characteristics of different kinds of bread during staling of the packaged bread were investigated. A linear dependence of change of average reflectance with time of storage of bread packed in polyethylene packaging was found. The highest values of average reflectance and dominant wavelength were found for barley bread (immediately after preparation), so it can be said that this sample was “conditionally” the lightest. During the whole keeping time, the average reflectance was the lowest in diet bread, so it can be “conditionally” considered as the darkest product. Colors of all investigated bread samples were lighter after three days of storage, compared to day 0. This is the consequence of staling and it was the most evident for the rye bread samples, probably because of high wheat flour levels in its formulation. Color qualities of the mentioned bread kinds depend on technological qualities and raw material compositions of bread (flour), so that color measurements could be used for initial screening of raw materials and processes for the production of the “anti-staling” breads.

## Figures and Tables

**Table 1.** Results of instrumental color determination of different kinds of bread immediately after production.

	***Measured and calculated values***							
**Bread type**	***CIE***				***CIELab***			
	***y* (%)**	***λ* (nm)**	***P* (%)**		***L***	***a***	***b***	**Δ*H_ab_***
Whole meal bread	37.79	569	29.16		67.86	−12.31	28.03	4.54
Rye bread	40.80	566	28.00		70.03	−13.79	27.59	4.23
Barley bread	48.99	572	27.66		75.44	−7.79	25.11	4.87
Diet bread	30.85	570	36.17		62.38	−9.59	28.36	5.04
	***Measured and calculated values***							
***Bread type***	***ANLAB***				***Hunter***			
	***L***	***A***	***B***	**Δ*E****_an_*	***L****_Hu_*	***a****_Hu_*	***b****_Hu_*	**Δ*E****_Hu_*

Whole meal bread	61.56	−11.65	26.12	35.95	61.47	−10.59	20.46	36.08
Rye bread	63.61	−13.10	25.85	34.93	63.87	−11.98	20.56	34.67
Barley bread	67.74	−7.47	23.83	28.82	69.99	−7.08	19.84	28.44
Diet bread	56.43	−8.97	26.08	38.82	55.54	−8.01	19.67	40.07

**Table 2.** Results of instrumental color determination of different kinds of bread after their storaging for 3 days at ambient temperature (20 ± 2 °C).

	***Measured and calculated values***							
**Bread type**	***CIE***				***CIELab***			
	***y* (%)**	***λ* (nm)**	***P* (%)**		***L***	***a***	***b***	**Δ*H_ab_***
Whole meal bread	46.26	557	26.32		73.71	−28.95	24.68	1.72
Rye bread	54.35	543	20.27		78.76	−32.95	23.88	1.17
Barley bread	55.18	540	16.88		79.14	−33.84	23.01	0.93
Diet bread	39.83	560	23.08		69.34	−29.00	25.08	1.78
	***Measured and calculated values***							
***Bread type***	***ANLAB***				***Hunter***			
	***L***	***A***	***B***	**Δ*E****_an_*	***L****_Hu_*	***a****_Hu_*	***b****_Hu_*	**Δ*E****_Hu_*

Whole meal bread	67.09	−27.59	23.35	38.96	68.01	−24.75	19.31	37.26
Rye bread	71.81	−31.54	22.79	39.65	73.72	−28.72	19.46	37.09
Barley bread	72.27	−32.47	21.98	39.78	74.28	−28.57	18.91	37.21
Diet bread	62.96	−27.41	23.49	40.97	63.11	−24.09	18.92	39.81

**Table 3.** Mathematical model of average reflectance as function of storage time.

**Mathematical model**	**y = ax + b**		
**Chi-sqr**	**0.1542**		
**Sample**	**a**	**b**	**Correlation coefficient**
Whole meal bread	2.84 ± 0.17	37.72 ± 0.33	0.99
Rye bread	4.48 ± 0.17	40.54 ± 0.33	0.99
